# Magnetic Field Distribution and Signal Decay in Functional MRI in Very High Fields (up to 9.4 T) Using Monte Carlo Diffusion Modeling

**DOI:** 10.1155/2007/70309

**Published:** 2007-10-02

**Authors:** Bernd Michael Mueller-Bierl, Kamil Uludag, Philippe L. Pereira, Fritz Schick

**Affiliations:** ^1^Max-Planck Institute for Biological Cybernetics, Spemannstraße 41, 72076 Tübingen, Germany; ^2^Department of Diagnostic Radiology, University Clinic Tuebingen, 72076 Tübingen, Germany; ^3^Section on Experimental Radiology, Department of Diagnostic Radiology, University Clinic Tuebingen, 72076 Tübingen, Germany

## Abstract

Extravascular signal decay rate R2 or R $2^{*}$ as a function of blood oxygenation, geometry, and field strength was calculated using a Monte Carlo (MC) algorithm for a wider parameter range than hitherto by others. The relaxation rates of gradient-recalled-echo (GRE) and Hahn-spin-echo (HSE) imaging in the presence of blood vessels (ranging from capillaries to veins) have been computed for a wide range of field strengths up to 9.4T and 50% blood deoxygenation. The maximum HSE decay was found to be shifted to lower radii in higher compared to lower field strengths. For GRE, however, the relaxation rate was greatest for large vessels at any field strength. In addition, assessments of computational reliability have been carried out by investigating the influence of the time step, the Monte Carlo step procedure, boundary conditions, the number of angles between the vessel and the exterior field ${\text{B}}_{0}$ , the influence of neighboring vessels having the same orientation as the central vessel, and the number of proton spins. The results were compared with those obtained from a field distribution of the vessel computed by an analytic formula describing the field distribution of an ideal object (an infinitely long cylinder). It was found that the time step is not critical for values equal to or lower than 200 microseconds. The choice of the MC step procedure (three-dimensional Gaussian diffusion, constant one- or three-dimensional diffusion step) also failed to influence the results significantly; in contrast, the free boundary conditions, as well as taking too few angles into account, did introduce errors. Next neighbor vessels with the same orientation as the main vessel did not contribute significantly to signal decay. The total number of particles simulated was also found to play a minor role in computing R2/ R $2^{*}$ .

## 1. INTRODUCTION

The effect of diffusion on signal decay in blood oxygenation level-dependent (BOLD) imaging is mainly due to the extravascular contribution of spins, especially at high field strengths >4 T [1]. In the current study, the well-known Monte Carlo (MC) approach modeling Brownian diffusion of protons in a background magnetic field has been used to compute extravascular (EV) BOLD signal changes. To this end, the static dipole model presented previously [2] has been extended to a dynamic model describing the sampling of phases of the individual protons moving in the inhomogeneous magnetic field.

Earlier studies on the effect of subvoxel variations in magnetic susceptibility were reported by Fisel et al. [3]. Weisskoff et al. compared MC simulations with experiments with polystyrene microspheres to demonstrate that enhanced relaxation can be explained quantitatively for both spin-echo and gradient-echo experiments [4]. The effect of an endogenous paramagnetic agent (deoxygenated hemoglobin) on image contrast has been addressed by several authors, for example, Ogawa et al. [5], Kennan et al. [6], and Boxerman et al. [7]. All models are based on the fact that in the vicinity of capillaries and venules, local magnetic field distortions are generated by the presence of paramagnetic deoxyhemoglobin in the blood.

Data from the models in the literature so far have mostly been restricted to a magnetic field strength of 1.5 T, that is, the clinical scanner field strength in the past, and mostly for GRE only. However, nowadays, scanners with high or ultra-high field strength for humans up to 9.4 T are available for research, and EV-BOLD data for these field strengths both for GRE and HSE have not yet been provided. The aim of the present work, therefore, was to investigate these issues at such high magnetic field strengths.

To examine the contribution of extravascular spin in isolation from other factors, an impenetrable vessel wall boundary for extravascular spins was assumed. The range of investigated susceptibility values was determined using a deoxygenation content of 5% at 1.5 T as the lowest susceptibility value and up to 50% at 9.4 T as the highest value. In addition, standard approaches used in the literature have been evaluated as to how they influence the computed relaxation rates. In particular, the choice of the time step, the diffusion step, the number of angles and the influence of neighboring vessels, and the number of protons were examined.

## 2. THEORY

Our aim was to study signal decay due to the phase sampling of the individual spins during their random movement. The spins in the brain parenchyma are diffusing in a background magnetic field caused by deoxygenated blood present in capillaries, venules, and veins. The field distribution is therefore determined by vessels inside a computational volume, filled with deoxygenated blood. The susceptibility creating the field distribution around the blood vessel is proportional to the level of blood deoxygenation and to the exterior field.

Weisskoff et al. proposed generalizing their results obtained using a numerical model by the use of the Bloch-Torrey equation [4]. Fujita also established a dimensionless equation which is ruled by two parameters [8]. We briefly recapitulate their arguments in the following paragraph and thereby show how their theories relate to one other.

Because the MC method solution must respect the Bloch-Torrey equations, generalized scaling laws might be derived in advance to generalize the numerical solutions [4]. If the length scale is made dimensionless by x↦y=λ⋅x , we obtain

(1) $$\frac{dS\left(y,t\right)}{dt}=i\omega \left(y\right)\cdot S\left(y,t\right)+\left(\lambda^{2}D\right)\cdot \nabla^{2}S\left(y,t\right).$$

If the time scale is made dimensionless by $t\mapsto t^{\prime }=t/\gamma$ , we obtain

(2) $$\frac{dS\left(x,t^{\prime }\right)}{dt}=i\cdot \gamma \cdot \omega \left(x\right)\cdot S\left(x,t^{\prime }\right)+\left(\gamma \cdot D\right)\cdot \nabla^{2}S\left(x,t^{\prime }\right).$$

Substituting λ by λ↦1/R and γ by γ↦ TE, the general scale independent relation

(3) $$\frac{dS}{dt}=-i\cdot \alpha \cdot S+\beta \cdot \nabla^{2}S$$

with α=ω⋅TE and $\beta =D\text{T}\text{E}/R^{2}$ can be established. From this equation, (3), (1), and (2) follow as special cases. The minus sign in (3) indicates the direction of rotation in the complex plane and therefore can be omitted. In the present article, we use cgs units with D=1 *μ*m ² /ms and TE = 40 milliseconds.

## 3. MATERIAL AND METHODS

### 3.1. Flow chart

**Figure 1:**
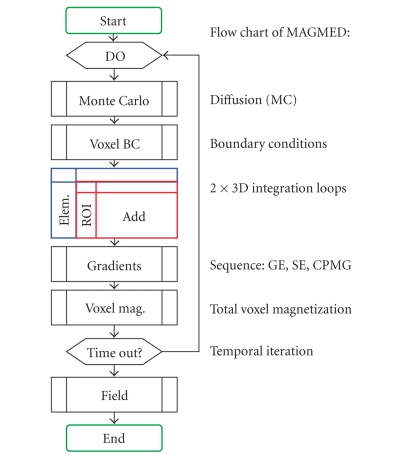
Flow chart of the model. It consists of the dipole model and a Monte Carlo time-step procedure. The inner of the two 3D loops is substituted in the present work by a known field distribution for the element (i.e., the field distribution for an infinite vessel).

Our model is similar to that of Boxerman et al. [7]. A flow chart illustrating the model is shown in Figure 1. The total signal is computed from individual complex transverse magnetizations of the protons, which are accumulating phases in the magnetic background field. The magnetic background field is described either by an analytic formula, or by integrating the individual contributions from the discretized susceptibility distribution.

We used the field distribution caused by a vessel segment approximated by a paramagnetic cylinder with infinite length to be the susceptibility distribution for an infinite vessel, which is given by

(4) $$\begin{array}{l} \delta \omega \cdot f\left(x\right)=\delta \omega \cdot {\left(\frac{1}{r}\right)}^{2}\cos\left(2\cdot \varphi \right)\sin2\left(\theta \right)\;\;\;\;\left(r\geq 1\right)\\ \;=\delta \omega \cdot \left(\cos^{2}\theta -\frac{1}{3}\right)\;\;\;\;\left(r\geq 1\right), \end{array}$$

where θ is the angle between the cylinder and the direction of the static magnetic field, *r* and φ represent the nondimensional radial coordinate relative to the vessel radius R and the azimuthal angle in the plane orthogonal to the cylinder, respectively, and δω is the susceptibility-induced maximal frequency shift occurring at the vessel surface, given by

(5) $$\delta \omega =2\pi \cdot \Delta \chi \cdot \omega_{0}\text{Hct}\left(1-Y\right).$$

In (5), Hct denotes the haematocrit value, that is, the fraction of the volume taken up by the red blood cells, Δχ is the volume susceptibility difference per unit Hct between fully oxygenated and fully deoxygenated blood, $\omega_{0}$ is the static magnetic field strength in terms of frequency, and *Y* is the degree of blood oxygenation.

Our parameter space was given by a haematocrit value of Hct = 0.4, a susceptibility of fully deoxygenated blood of Δχ= 0.18 ppm (cgs units). Frequency thus varied from 1.4454 Hz (corresponding to 1−Y = 5% at 1.5 T) to 65 × 1.4454 Hz (90.58 Hz corresponding to 1−Y = 50% at 9.4 T) in steps of 5.7816 Hz. The radii varied from 3 *μ*m to 60 *μ*m in logarithmical equidistant values.

Alternatively, one could discretize the paramagnetic cylinder representing the vessel into elementary volumes, for example, of size of the computational cell. In the dipole model, to each elementary volume, a dipole strength $p_{m}=M\Delta V$ is assigned, where *M* is the magnetization proportional to the exterior field strength $M=\chi B_{0}$ . The elementary volume then contributes to the field distribution by an amount of $\Delta B_{z}=\left(p_{m}/r^{3}\right)\left(3\cos^{2}\theta -1\right)$ .

To compute the total field distortion *B*_*z*_, a spatial integration to summate all contributions has to be performed. Diffusion was computed according to 3 models which can be distinguished by the choice of the diffusion step. They are given by the following definitions.

**STEP1D**

Take a fixed step ( $\ell =\sqrt{2D\tau }$ ) in each direction (along the positive or negative axis, directions chosen randomly).

**STEP3D**

Take a fixed step ( $\ell =\sqrt{6D\tau }$ ) in a randomly chosen direction.

**GAUSS3D**

Take a random step with a Gaussian distribution ( $\sigma =\sqrt{6D\tau }$ ) in a randomly chosen direction.

In the formulae, D is the diffusion coefficient and τ is the time step. As diffusion strength, we used D=1 *μ*m ² /ms, which is typical for the cerebral cortex [7]. As a random generator, the routine ran1 from the numerical recipes [9] was chosen. The various MC step methods were first tested by computing the radial distribution of the protons after N time steps for M protons. Unless stated otherwise, the time step τ was chosen as 10 microseconds and TE was 40 milliseconds at maximum, and the number of protons was 24^3^.

Using these definitions, the routines given by Ogawa et al. [5] (Step3D), Boxerman et al. [7] (Gauss1D), and Weisskoff et al. [4] (Gauss1D) were explored. Gauss1D, of course, is a hybrid of Step1D and Gauss3D. The arbitrarily chosen direction must be determined from random values lying inside a sphere (except the origin). To determine the direction, the values have been projected onto the unit sphere's surface. The time step was chosen as either 10 microseconds or 200 microseconds for the GE and SE signal relaxation rate computations. Initial positioning and the Monte Carlo steps were ruled by two separate random generators from Press et al., that is, using the same starting conditions, the results can be reproduced completely. Computations have been repeated and mean values have been calculated to reduce numerical noise due to the finite-sized sample of proton spins.

The model consists of the following steps:

1. place a cylinder with a given orientation relative to B_0_ in a volume of interest;
2. distribute protons randomly in that volume of interest;
3. compute the field at the location of each proton as a superposition of the field generated by the cylinder;
4. (optional) add the gradient fields from the MR sequence at the location of each proton (e.g., to simulate a CPMG echo train);
5. advance the phase of each proton according to its local field and, in case of a 180° pulse for HSE, invert the direction of phase accumulation;
6. advance each proton in an MC step in an arbitrary direction (in the case of a 3D step chosen from an arbitrary position on the surface of a unit sphere);
7. if the proton has transgressed the cylinder wall, repeat step (6) with a given probability, which is determined by the vessel wall porosity (in our case: repeat step (6) always);
8. if the proton has left the volume of interest, it might reenter, depending on suitable boundary conditions.

The resulting signal was computed by summating all (normalized) complex magnetizations of the EV spins. Spins which did not contribute (spins inside the vessel) were ignored.

In (4), the cosine of the angle is computed as a scalar vector product. This, together with expressing the trigonometric functions by a cosine function, prevents the evaluation of trigonometric series, which is highly time consuming.

Instead of one cylinder, we also used 5 and 9 cylinders as illustrated, for example, by Kennan et al. for 5 cylinders [6].

As conditions at the limits of the computational domain, we used periodical boundary conditions (with the spins re-entering the computational domain from the adjacent side of the domain), reflecting boundary conditions (with the spins being reflected at the limits of the domain), and free boundary conditions (where the spins are free to leave the domain).

Background gradients can be added to the exterior field at any time during the computations, so that diffusion-sensitive sequences like CPMG can be established.

The occurrence of vessel direction varies with sin⁡(θ) , where θ is the angle between the exterior field and the vessel orientation. For our computations, we used 6, 9, and 18 angles, equally distributed between 0 and 90°. The total signal from vessels with varying orientation thus becomes [5]

(6) $$S_{\text{t}\text{o}\text{t}}=\frac{\sum \sin\left(\theta \right)\cdot \left\|S\left(\theta \right)\right\|}{\sum \sin\left(\theta \right)}.$$

The relaxation rate R2 was computed using a two-point evaluation function at TE = 16 milliseconds and TE = 40 milliseconds assuming $S_{\text{tot}}=S_{0}^{*}\exp\left({\text{-TE}}^{*}\text{R2}\right)$ [5].

## 4. NUMERICAL RESULTS

**Figure 2:**
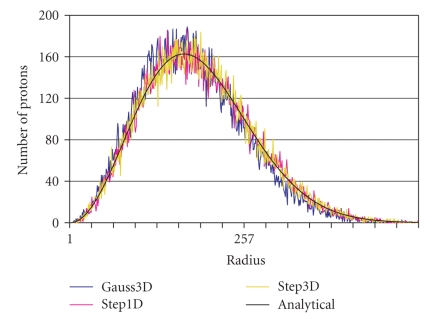
The radial spin position distribution shown after diffusion at time interval TD. The protons all started from the origin at T = 0 and propagated in space corresponding to the different Monte Carlo step procedures Step1D, Step3D, and Gauss3D.

**Figure 3:**
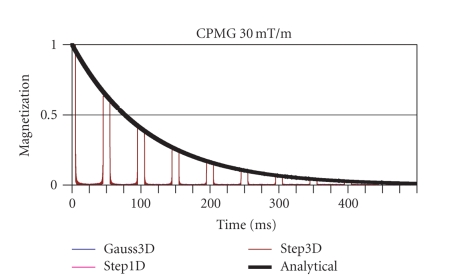
The analytical versus the computed decay curve of the transverse magnetization shown for a CPMG set of parameters.

Testing of the diffusion process is shown in Figures 2and 3: Figure 2 shows the distribution of spins after a fixed diffusion time TD together with the theoretical distribution. Figure 3 shows the CPMG signal decay together with its analytical course. Both the local spin distribution as well as the CPMG signal decay follows the theoretical predictions with some random noise due to the limited number of protons.

**Figure 4:**
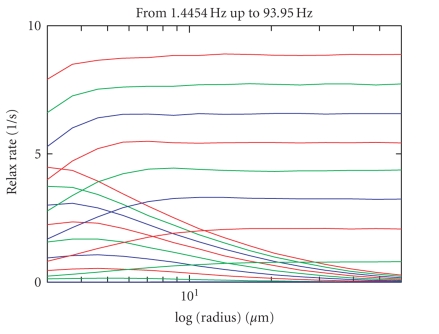
R2/R2* relaxation rate (in *s* ^−1^) for SE, GE, from deoxygenation corresponding to a frequency of 1.4454 Hz to 65 × 1.4454 Hz. Data are shown in steps of 8 × 1.4454 Hz. The time step length in these computations was 10 microseconds. The mean value for 8 computations with different random series initialization is shown.

Results for the computation of R2/R2* for GRE and HSE imaging are shown in Figure 4. The curves are the mean values of 8 computations, each with a different random series initialization. Time step length has been set at 10 microseconds within these simulations. As can be seen, HSE relaxation rates are greatest for small radii, whereas for GRE large vessel radii have the highest relaxation rates. In addition, as expected, the relaxation rate both for GRE and HSE increase with blood oxygenation and field strength. Higher susceptibility for HSE results in shifting the maximal relaxation rate to even smaller radii.

**Figure 5:**
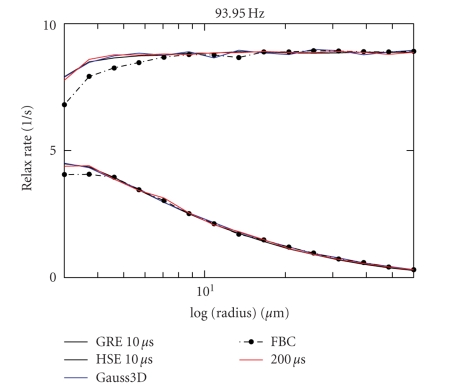
R2/R2* relaxation rate (in *s* ^−1^) for time step length of 10 microseconds versus 200 microseconds. Data for the Gauss3D step and for the free boundary conditions are also shown. The free boundary condition data clearly deviate from the other data.

Comparison of these results to computations with a time step length of 200 microseconds is shown in Figure 5. They agree with the results using the smaller time steps. The 200-microsecond data are slightly noisier since there is no averaging as in the 10-microsecond data, which have been computed 8 times for different random series initialization. Comparisons to a Gauss3D diffusion step computation and to a free boundary condition computation are also shown (time step 200 microseconds in both). Only the data for the free boundary computation are slightly inconsistent with the others.

**Figure 6:**
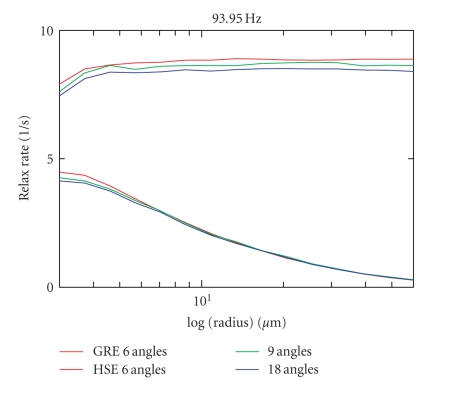
R2/R2* relaxation rates (in *s* ^−1^) shown for 9 angles and 18 angles compared to the data for 6 angles between the exterior field and the vessel direction.

Figure 6 compares the results of the 8 computations with a computation using 9 or 18 angles instead of 6 (time step 200 microseconds each). A nonnegligible deviation of the GE curve, and a slightly smaller deviation also for the SE curve can be seen (in the static dephasing regime: a deviation of 5% for 6 angles, i.e., a deviation of 2.5% for 9 compared to 18 angles). Again, data from the 9-angle versus 18-angle comparison have been computed only once.

**Figure 7:**
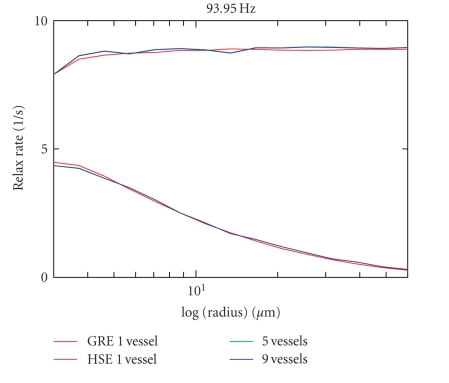
R2/R2* relaxation rates (in *s* ^−1^) shown for 5 vessels (central vessel and 4 next neighbors), respectively, shown for 9 vessels (8 next neighbors) versus data for one single vessel. All neighbor vessels possess the same direction as the central vessel.

In Figure 7, neighbor rods are taken into account and the result is compared to the result of the 8 computations with different random series initialization. Data for the 5 or 9 next neighbors have been computed once. There are no apparent differences compared to the computations with only one vessel.

**Figure 8:**
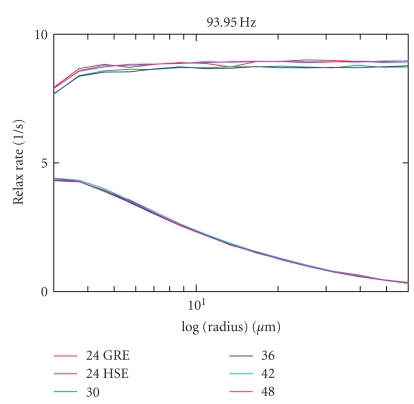
R2/R2* relaxation rates (in *s* ^−1^) shown for the computational set up with edge lengths of the computational cell of 24, 30, 36, 42, and 48 mean spin-to-spin distances along each edge, corresponding to 24^3^, 30^3^, 36^3^, 42^3^ and 48^3^spins in each computational cell.

Finally, in Figure 8, the data for different numbers of spins in the computational domain can be found. The numbers of spins distributed in the computational domain were 24^3^, 30^3^, 36^3^, 42^3^, and 48^3^ (time step 200 microseconds each). A change in the number of spins does not result in any significant difference between the calculated relaxation rates.

## 5. DISCUSSION

When compared to lower field strengths, the maxima of the spin-echo decay were found to be shifted to lower radii. A set-up with too few angles as well as the use of free boundary conditions introduces errors and therefore should be avoided. All other parameters (time-step, number of protons, and influence of neighboring vessels of the same orientation, choice of the MC step model used) did not influence the results in our computations.

The current loop approach according to Biot Savart proved the use of the dipole formulae for a finite voxel element to be correct [2]. The plot of the field distribution of an infinite vessel is in agreement with the field distribution of a very long discretized vessel. However, we found that results for a discretized cylinder slightly deviate from the results for an infinitely long cylinder. For consistency, we show only results obtained for the infinite cylinder in the present work. Moreover, the CPMG decay experiment showed that our diffusion modeling works quite well for all MC step methods investigated.

As discussed by Ogawa et al. [5], at the limit of infinite numbers of spins and time steps, the signal S(θ) in (6) would be real. However, because we use a finite number of proton spins, there is a residual complex part of the signal. In (6), we forced *S* to be real by taking its absolute value. However, as the number of spins increases, the residual part should diminish and, at the limit of infinite numbers of protons, should vanish.

The precursor modeling approaches according to Balac et al. [10], Bhagwandien et al. [11], Lüdeke et al. [12], and Bakker et al. [13] are all limited in that they rely either on singular analytical solutions for spheres and cylinders, or on complicated procedures such as boundary element methods (BEMs), finite differences (FDs), or finite element methods (FEMs). The dipole model, in contrast, uses arbitrarily chosen elements (discretized to a grid) in combination with simple geometries (spheres, finite cylinders, parallelepipeds). This allows the design of special interventional instruments (e.g., a biopsy needle with markers) and the computation of the decay of the transverse magnetization in “real world” geometry as in a trabecular bone model, or in a vessel network to model signal decay in brain parenchyma. Branches of vessels might be simulated using our model. Use of analytic field distributions remains possible, thereby allowing modeling similar to Boxerman et al. [7], Ogawa et al. [5], Kennan et al. [6], or Fujita [8].

Discretization of the susceptibility distribution can be made more precise than the mean distance between protons by introducing distance factors [14]. An estimate of arbitrarily high resolution can then be achieved by Richardson Extrapolation [9].

Intra- and extravascular pools were separated by a routine which tests whether a spin is intra- or extravascular. The same routine can be used to detect the transgression of a boundary, and in the case of impenetrable vessel walls, the diffusion step can then be rejected until a transgression-free step has been attained. This is achieved by testing whether a spin was extravascular before the MC step but becomes intravascular after it, or vice versa. Actually, vessel walls are to be regarded as impenetrable, because exchange rates are much longer (typically 500 milliseconds) than TE (typically 100 milliseconds) as discussed in Fisel et al. [3], and in Boxerman et al. [7]. Using a rejection with probability *P* (drawn from a random series), vessel walls might be modeled as partially penetrable. Spins belonging to the intravascular pool are not considered to contribute to the BOLD signal.

The boundaries of the computational domain might be treated as periodical boundary conditions, reflection of spins at the boundaries or by no conditions at all. However, we observed that tests with such penetrable boundaries (also called “free boundary conditions”) lead to slightly false results.

Let us summarize an important, specific finding revealed in Figure 4 of Section 3. At high field strength (9.4 T), spin-echo imaging is especially sensitive at about 3 *μ*m radii. The maximum of the spin-echo decay is shifted to lower radii than with lower field strengths. This makes spin-echo imaging especially sensitive to small vessels and capillaries.

The computation with 18 angles revealed a source of discretization error. The 6 angles used regularly in our computations essentially yield only an approximate result. However, since the effort to compute with 18 angles is 3 times greater, we have restricted our analysis to 6 angles. One should bear in mind that this leads to a (somehow limited and therefore minor) error in R2/R2*. Moreover, we found that placing vessels in the next 6 or 9 neighbor volumes does not change the result. Vessels with orientations different from the orientation of the central vessel in the next neighbor volumes have not been investigated and are left for future work.

From 24^3^ protons on, the result does not change significantly with increasing numbers of protons. However, to obtain smoother curves (less numerical noise due to a limited number of protons), computations might be repeated using different random initializations for the MC step procedure. Computing time largely depends on the hardware resources available. For the computations with TE = 40 milliseconds in steps of 10 microseconds, computing the complete parameter-space for one random series initialization value with 24^3^ protons took approximately 8 days on a Pentium machine with a clock rate of 1.8 GHz.

The model might be further improved by introducing Bloch's equations instead of considering a constant transverse magnetization. Pulses and gradients can then be simulated to model a real pulse sequence with all its implications regarding magnetization. The diffusion constant might also be replaced by a diffusion tensor, making the treatment of problems based on diffusion anisotropy possible.
